# Advancing Our Understanding of Psychological Stress and Coping Among Parents in Organized Youth Sport

**DOI:** 10.3389/fpsyg.2019.01600

**Published:** 2019-07-12

**Authors:** Chris G. Harwood, Sam N. Thrower, Matthew J. Slater, Faye F. Didymus, Lucy Frearson

**Affiliations:** ^1^School of Sport, Exercise and Health Sciences, Loughborough University, Loughborough, United Kingdom; ^2^Department of Life Sciences, University of Roehampton, London, United Kingdom; ^3^School of Life Sciences and Education, Staffordshire University, Stoke-on-Trent, United Kingdom; ^4^Institute for Sport, Physical Activity and Leisure, Leeds Beckett University, Leeds, United Kingdom

**Keywords:** sport parents, stressors, appraisal, emotion, coping, coping effectiveness, mixed method

## Abstract

The current study investigated psychological stress among parents of competitive British tennis players. Adopting a multipart concurrent mixed method design, 135 British tennis parents completed a cross sectional online questionnaire to examine their primary appraisals, emotions, and coping strategies associated with self-disclosed stressors. Hierarchical content analysis was conducted on open ended questionnaire responses to identify key stressors and coping strategies, and descriptive and inferential statistics were utilized to explore the differences between various components of the process. The findings revealed a range of organizational, competitive, and developmental stressors. These stressors were predominantly appraised as harm or challenge, and anxiety and anger were the most prominent emotions that the parents experienced. Statistically, parents experienced greater anger in relation to competition (compared to organizational and developmental) stressors, whilst harm appraisal increased negative emotions, and challenge appraisal increased positive emotions. Findings also highlighted how parents used a number of mastery, internal regulation, and goal withdrawal coping strategies, which varied statistically in degrees of reported effectiveness. The contribution of these findings to the stress literature and their applied implications are discussed.

## Introduction

Psychological stress among athletes and coaches has been well documented in the sport psychology literature (e.g., Didymus and Fletcher, 2012; Didymus, 2017). Transactional and relational theories of stress are some of the most widely used and tested in sport [see e.g., transactional stress theory; Lazarus and Folkman, 1984; cognitive-motivational-relational theory (CMRT) of stress and emotion; Lazarus, 1999, 2000]. These theories posit that stress is a transaction between an individual and his or her environment, and that individuals appraise stressors in relation to their goals, values, and beliefs. According to the CMRT (Lazarus, 1999, 2000), appraising is an evaluative process during which individuals construct relational meanings about the stressors they encounter. Relational meanings may relate to challenge, threat, harm, or benefit, and each has different implications for emotions, coping, and other outcomes (e.g., well-being and performance). If a stressor is appraised as relevant to an individual, coping will ensue. The degree to which coping optimizes stress transactions is known as coping effectiveness. Drawing on transactional theories, researchers have used qualitative and quantitative methods to unearth stressors (i.e., competitive, organizational, and personal) that athletes experience (e.g., Nicholls et al., 2005), the ways in which they are appraised (e.g., Didymus and Fletcher, 2012; Doron and Martinent, 2017), the emotions experienced (Nicholls et al., 2011; Moore et al., 2012), and the strategies used to cope (see, for a review, Nicholls and Polman, 2007). Researchers have also, more recently, started to explore the relationships and interactions between different components of stress transactions (e.g., stressors, appraisal, emotion, and coping; Nicholls et al., 2012; Doron and Martinent, 2017; Gomes et al., 2017).

Although the stress literature has predominantly focused on the experiences of athletes and coaches, researchers have offered initial understanding of the stressors associated with parenting in youth sport (Harwood and Knight, 2009a, b; Harwood et al., 2010). Initiating this line of inquiry, Harwood and Knight (Harwood and Knight, 2009a, b) explored the stressors that British tennis parents experienced at different stages of their children’s development. Data were collected during these studies via 123 open-ended surveys and 22 semi-structured interviews with tennis parents. Parental stressors centered on the organizational aspects of children’s tennis (e.g., injuries, finances, and time), competition demands (e.g., watching matches, players/opponents cheating, and limited effort), and developmental concerns (e.g., players’ future in tennis and transitional decisions regarding schooling). Taken together, these findings illustrate how parents’ experiences are influenced by the nature of the sport, the sport organizational system, and their children’s developmental stage. This supports the notion that stress is a context-dependent and temporal process (Lazarus, 1999).

Alongside this body of work, a small number of studies have explored the emotions that parents experience in youth sport settings (e.g., Goldstein and Iso-Ahola, 2008; Omli and LaVoi, 2012). For example, Omli and LaVoi (2012) identified the sources of anger for parents at youth sport competitions based on the assumption that anger fuels negative parental behavior at sporting events. Surveys with 773 parents of young athletes (aged 5–19 years) revealed that 98% of participants had experienced anger during sport competitions and that the parents experienced stressors such as the unjust, uncaring, and incompetent behaviors of coaches, other parents, officials, and athletes. Whilst anger appears to be a particularly salient emotion experienced by parents in youth sport, research has also illustrated how parents often feel disappointment (Wiersma and Fifer, 2008; Dorsch et al., 2009) and embarrassment if their child is underperforming or behaving poorly (Dorsch et al., 2009; Harwood and Knight, 2009a, b). Although these studies have offered initial insight into emotions among sport parents, it is not clear how parents’ appraisals shaped their emotional responses or the types of appraisals that parents experience. Furthermore, very little is known about the other theoretically proposed emotions that parents may experience when watching their children compete (e.g., anxiety, dejection, happiness, and excitement; Jones et al., 2005). Developing a more robust body of evidence in this area is important if we are to understand the psychological processes that determine parents’ experiences of stress and, in turn, the behaviors that they exhibit and the support (or lack thereof) that they are able to offer to their children (cf. Webster-Stratton, 1990; Lazarus, 1999).

Many approaches to the classification of coping have been proposed (see e.g., Lazarus and Folkman, 1984; Connor-Smith et al., 2000; Skinner et al., 2003; Gaudreau and Blondin, 2004) yet a consensus on how best to classify coping is yet to be reached. To address this challenge, Nicholls et al. (2016) reviewed the strengths and limitations of various approaches and, during a meta-analysis, found support for a three-factor classification system (i.e., mastery, internal regulation, and goal withdrawal). Despite other approaches to coping classification holding promise, this three-factor approach appears useful for enhancing conceptual clarity and informing future research. There is limited empirical research that has specifically set out to explore the coping strategies that parents use. One notable exception is a recent study by Burgess et al. (2016) who used interpretive phenomenological analysis (IPA) to examine how parents (*n* = 7) of elite youth gymnasts cope with stressors. Their findings suggest that the parents encountered a variety of competitive, organizational, and developmental stressors and employed a range of coping strategies, including detaching, normalizing experiences, having a willingness to learn, and managing emotional reactions (Burgess et al., 2016). This study provided initial insight to the coping strategies employed by youth sport parents and suggests that parents utilize multiple strategies in combination when attempting to cope with stressors. Nevertheless, it is not clear which strategies are effective for parents. As Nicholls (2016) suggested, understanding more about coping effectiveness and the factors that influence coping (i.e., stressors, appraisals, and emotions) would help applied researchers and practitioners to develop more effective and tailored interventions (see Thrower et al., 2017).

Considered collectively, the aforementioned studies have offered preliminary understanding of different components of psychological stress transactions among sport parents. Nevertheless, by focusing on the discrete components of transactions, these studies have overlooked the crucial associations between stressors, appraisals, emotions, and coping that have implications for parents’ behavior, health, and well-being (Lazarus and Folkman, 1984; Lazarus, 1999). In addition, extant literature almost entirely overlooks the concept of appraising among parents, which is problematic given the central role that it has in determining the outcomes of stress transactions. There is clearly a need to go beyond fragmented studies and give more holistic consideration to stress transactions. As Harwood and Knight (2009b) suggested, “future research should pay closer attention to understanding the full stress and coping process in sport-parents to furnish practitioners, parents, and organizations with more precise intervention ideas, education, and skills” (p. 34). Such studies represent a significant methodological challenge for researchers due to the contextual nature of stressors and the absence of measures that capture the complexity and idiographic nature of stressors and coping. With this in mind, Nicholls (2016) suggested that exploring the nuanced ways in which individuals cope with stressors might require more sophisticated and novel research designs. The aim of this study was, therefore, to build on the aforementioned sport parent research and explore more fully psychological stress among parents of competitive British tennis players. To achieve this aim, the current study used a multipart mixed method design to answer a series of interconnected questions: (a) What are the prominent stressors that tennis parents experience? (b) How are these stressors appraised? (c) What emotions are associated with such stressors? (d) What coping strategies do parents use? (e) How effective are these coping strategies? and (f) Does perceived effectiveness vary as a function of stressor type, appraisal, and coping strategy used?

## Methodology and Methods

### Research Design

This study was conducted from a post-positivistic philosophical position which acknowledges that some aspects of the social world cannot be directly measured but seeks to retain an objective approach that is free from bias (Weed, 2009). As such, post-positivists loosen the strict positivistic belief in value-free inquiry, yet still test theories, often quantify their data, and adopt traditional evaluation criteria (Krane and Baird, 2005; Denzin and Lincoln, 2011). Consistent with this philosophical position, a novel multipart concurrent mixed method design (Morse, 2003; Morgan, 2013) was adopted in which multiple qualitative and quantitative data were collected simultaneously within one study to answer the research questions. Both types of data were collected at the same time and were given equal emphasis and priority (i.e., QUAL + QUANT; Morse, 2003). Specifically, a cross-sectional online questionnaire was used where qualitative components were included to identify prominent stressors and coping strategies (Harwood and Knight, 2009a; Burgess et al., 2016) and quantitative elements captured primary appraisals, emotions, and coping effectiveness (Jones et al., 2005; Hanton et al., 2012). Qualitative data was subsequently transformed (i.e., quantified) to allow for combined analyses to be conducted which in turn enabled the data to be merged and interpreted within both the results and discussion sections.

### Participants and Sampling

Following institutional ethical approval, homogeneous purposeful sampling was used to recruit parents of British junior tennis players (aged 5–18 years). An email invitation was sent from the national governing body (i.e., the Lawn Tennis Association) to parents of an estimated 1,500 British tennis players who met the selection criteria (i.e., parents of children who regularly participate in tennis between the ages of 5–18 years). One hundred and thirty five parents (41 men, 93 women, one parent chose not to report gender) responded to this invitation and agreed to participate in the study (9.0% response rate). Most participants identified as being their child’s biological parent (97.77%), were in a relationship (94.77%), and had between 2 and 17 years (*M* = 6.88; *SD* = 3.07) experience as a tennis parent. Participants’ children were between the ages of 6 and 18 years of age (*M* = 12.29; *SD* = 2.52) and were predominantly male (65.18%). Thirty-six participants’ children played ‘mini tennis’ (5–10 years) and had ratings between Red 4 (starting/lowest possible rating) and Green 1^*^ (the best/highest possible rating) specific to ‘mini-tennis’. Ninety-nine participants’ children played junior tennis and had ratings between 10.2 (starting/lowest possible rating) and 2.2 (*M* = 6.13; *SD* = 1.98). The highest (i.e., best) possible rating in British Tennis across junior and adult players is 1.1.

### Procedure

Those parents who responded favorably to the invitation were emailed a link to the online questionnaire where participant information for informed consent was provided. Parents who consented to participate at this point were then provided with an introductory guide containing instructions on how to complete the questionnaire. This guide was designed to enhance parents’ understanding of psychological stress, define key terms (e.g., stressors, primary appraisals, emotion, coping, and coping effectiveness), and provide worked examples. Once parents had read the introductory guide, they were asked to record up to five of the most prominent stressors that they had faced as a tennis parent. Participants were then asked to record the ways that they appraised each stressor (i.e., harm/loss, threat, challenge, and benefit; Lazarus, 1999) and the emotions associated with it (i.e., anger, dejection, anxiety, happiness, and excitement; Jones et al., 2005). Finally, parents were asked to record up to three strategies that they used to cope with each stressor in an open-ended format and how effective they considered each strategy to be.

### Qualitative Data Collection

In line with previous research (i.e., Harwood and Knight, 2009b; Levy et al., 2009; Burgess et al., 2016) a qualitative approach was used to identify the individual and subjective stressors that parents experienced and the ways in which they attempted to cope in their own words (Lazarus, 2000). Specifically, questionnaires with open-ended answer boxes were used to collect qualitative data about parents’ most pertinent stressors and associated coping strategies (Harwood and Knight, 2009b). Parents were given specific instructions about what to include in each open-ended box. For example, parents were asked to: “Record the coping strategies that you used in response to the stressor using the boxes below. Each coping strategy should go in a separate box. You can enter one or more (up to three) coping strategies depending on what you did to cope with that stressor.” Each open-ended question box offered unlimited space and parents were encouraged to provide as much depth and detail as possible.

### Quantitative Data Collection

#### Primary Appraisals

Drawing on the procedures outlined by Hanton et al. (2012), parents in the current study were asked to self-report whether they appraised each stressor as harm/loss (i.e., damage to goals, values, or beliefs that has already occurred), threat (i.e., future damage), challenge (i.e., anticipated gain), or benefit (i.e., gain that has already occurred; Lazarus, 1999). Parents were asked to select the most relevant primary appraisal for each stressor. The current study focused solely on primary appraisals because this part of appraising is thought to have salient implications for coping, emotion, performance, and well-being (see e.g., Lazarus, 1999; Didymus, 2017). Secondary appraising, which refers to evaluations of available coping resources, is also relevant as a determinant of emotional responses in Lazarus’ model. Nevertheless, given the complexity of the current design and anticipated analyses, an examination of this part of stress transactions was deemed beyond the scope of the current work.

#### Emotion

Based on the Sport Emotion Questionnaire (SEQ; Jones et al., 2005), which has demonstrated validity and internal consistency in a number of sport settings, participants were asked to record how anxious (i.e., uneasy, tense, nervous, apprehensive, or anxious), dejected (i.e., upset, sad, unhappy, disappointed, or dejected), excited (i.e., exhilarated, excited, enthusiastic, or energetic), happy (i.e., pleased, joyful, happy, or cheerful), and angry (i.e., irritated, furious, annoyed, or angry) they felt in response to each documented stressor. Specifically, participants were asked to record the extent to which they experienced each emotion on a 5-point rating scale of zero (not at all), one (a little), two (moderately), three (quite a bit), and four (extremely).

#### Coping Effectiveness

A coping effectiveness score was used to understand whether the strategies employed were perceived to be successful in alleviating the negative outcomes of stressors. In accordance with previous studies (Nicholls et al., 2007; Levy et al., 2009; Didymus and Fletcher, 2012), participants were asked to rate on an 11-point scale (0–10) how effective they perceived each individual coping strategy to be. For the purpose of this study, a perceived coping effectiveness score of zero was considered to be completely ineffective, a score of five was moderately effective, and a score of 10 was considered completely effective.

### Data Analyses

#### Qualitative Data

Qualitative data from the online open-ended questionnaire were analyzed using an abductive (i.e., inductive and deductive) approach to hierarchical content analysis. This approach has been applied elsewhere in the literature (e.g., Didymus, 2017) to encourage the creation of new ideas (i.e., inductive logic) whilst also applying theoretical frameworks or lenses to participants’ experiences (i.e., deductive logic). Although inductive reasoning was used to guide the initial stages of analysis, existing stressor (Harwood and Knight, 2009a, b) and coping classifications (Nicholls et al., 2016) were subsequently and deductively used to promote consistency in the terminologies that are applied within published literature. In line with the procedures outlined by Sparkes and Smith (2014), qualitative data were read and re-read to promote content familiarity and each open-ended response was labeled as a lower order theme. Following initial labeling, ideas that represented stressors or coping strategies reported by parents were grouped together to create meaningful higher order themes and general dimensions. Next, themes were crosschecked and thoroughly re-examined by the second named author and then confirmed by the first named author (Sparkes and Smith, 2014). Finally, tables were produced to reflect the hierarchical nature of the chosen method of analysis, including the frequencies of each cited stressor or coping strategy.

#### Quantitative Data

Quantitative data analysis started by calculating the overall frequency with which each type of appraisal was reported (i.e., harm/loss, threat, challenge, and benefit). Providing sufficient power (Clark-Carter, 2010), the total dataset for emotions was *n* = 342, and for coping effectiveness *n* = 646. Descriptive statistics (i.e., means and standard deviations) for the emotions experienced (i.e., anxiety, dejection, happiness, excitement, and anger) and coping effectiveness were then calculated. Data were screened for parametric assumptions and detected the presence of outliers in happiness and excitement variables. Accordingly, when analyzing the data, sensitivity analyses were conducted without these extreme cases (i.e., the removal of *n* = 4 each for happiness and excitement). These exclusions did not materially change the pattern of results. The main and interaction effects were the same, whilst two additional pairwise comparisons emerged as significant (these points are highlighted in the “Results” section). To complement the descriptive data, differences in emotions experienced as a function of stressor category and appraisal were explored using a 3 (stressor: competition vs. organizational vs. developmental) × 3 (appraisal: harm vs. threat vs. challenge) multivariate analysis (MANOVA). Differences in coping effectiveness as a function of stressor category, appraisal, and coping strategy were then assessed using a 3 (stressor: competition vs. organizational vs. developmental) × 3 (appraisal: harm vs. threat vs. challenge) × 3 (coping strategy: mastery vs. internal regulation vs. goal withdrawal) analysis of variance (ANOVA). Any significant main or interaction effects were followed up with Bonferroni adjusted pairwise comparisons.

## Results

In accordance with guidance from Lazarus (1999), the results section is organized by the components of psychological stress that were examined to provide a full and comprehensive view of the data. Data relating to stressors are presented first, followed by primary appraisals, emotions, coping strategies, and coping effectiveness. The final sub-sections of the results explore the statistical differences in emotions experienced and coping strategy effectiveness.

### Stressors

Data analysis generated three general dimensions of parental stressors: (a) organizational; (b) competitive; and (c) developmental. These three dimensions contained a total of 20 higher order themes, 51 lower order themes (see Table 1), and 342 individual raw data themes or stressors (eight stressors were not grouped into lower and higher order themes or dimensions due to limited relevance or coherence within the responses).

**TABLE 1:** The general dimensions, higher order themes, and lower order themes of stressors reported by parents (*n* = 135), including the frequency of which each was reported.

**General dimensions**	**Higher order themes**	**Lower order themes**	**Frequency**	
			***N***	***%***
Competition	Child’s opponent		33	24.44
		Bad line calls and cheating	24	17.78
		Aggressive or inappropriate behavior	9	6.67
	Child’s behavior		32	23.70
		Bad physical and verbal behavior	19	14.07
		Distress and limited emotional control	6	4.44
		Reluctance to challenge line calls/decisions	5	3.70
		Negative body language	2	1.48
	Other parents		21	15.56
		Bad behavior and attitude	9	6.67
		Interfering with play	7	5.19
		Intimidating and aggressive behavior	5	3.70
	Child’s performance		20	14.81
		Not playing to full potential	18	13.33
		Limited effort	2	1.48
	Watching a match		20	14.81
		Feeling nervous/worried about child’s performance	17	12.59
		Feeling helpless during a match	3	2.22
	Outcome of matches		16	11.85
		Consoling child/helping them to cope	7	5.19
		Child’s reaction to match outcome	4	2.96
		Child losing a match	3	2.22
		Spouse’s reaction to match outcome	2	1.48
	Child’s psychological readiness to perform		9	6.67
		Pressure/expectation that child places on themselves	7	5.19
		Child’s negative approach going into a match	2	1.48
	Poor refereeing		4	2.96
Organizational	Finances		34	25.19
		Cost of coaching, tournaments, and travel	29	21.48
		Financial impact on family and siblings	3	2.22
		Lack of player funding	2	1.48
	Time		30	22.22
		Limited family and partner time	13	9.63
		Time commitment	6	4.44
		Work/tennis role conflict	4	2.96
		Effect of unequal time spent on siblings	4	2.96
		Impact on social life/personal time	3	2.22
		Organization of tennis schedule	2	1.48
	Coaching and training		22	16.30
		Commitment, communication, and relations with coach	9	6.67
		Training program	6	4.44
		Specific disagreement with coach	4	2.96
		Access to training facilities	3	2.22
	Organizing bodies		20	14.81
		Lack of recognition and support	8	5.93
		Pressure of the rating system	6	4.44
		Problems with talent identification system	3	2.22
		Disorganization and management issues	3	2.22
	Tournaments		17	12.59
		Issues with entry, draws, and seedings	6	4.44
		Traveling to tournaments	5	3.70
		Poor organization/communication at tournament	3	2.22
		Lack of umpire present	2	1.48
		Tournament schedules	2	1.48
	Injury		9	6.67
		Overuse injury	5	3.70
		Fear of injury	2	1.48
		Limited knowledge regarding injuries	2	1.48
Developmental	Child’s progress in tennis		22	16.30
		Selection pressure	9	6.67
		Progression relative to peers	6	4.44
		Tennis rating	4	2.96
		Limited effort in training	3	2.22
	Tennis decisions		10	7.41
		Coaching decisions	4	2.96
		Tournament decisions	4	2.96
		Training decisions	2	1.48
	Child’s education and social development		5	3.70
	Child’s future in tennis		3	2.22
	Impact of tennis on other sports/hobbies		2	1.48
	Child’s wellbeing and happiness		2	1.48
Other			8	5.93

#### Competition Stressors

Eight higher order themes, 19 lower order themes (see Table 1), and 155 raw data themes were associated with junior tennis competitions. Specifically, ‘child’s opponent’ (24.44%) was the most prominent cause of stress for parents during competitions and, in particular, ‘bad line calls and cheating’ (17.78%) and ‘aggressive or inappropriate behavior’ (6.67%). As one parent explained: “I find it difficult watching the behavior of my daughter’s opponent (i.e., tantrums, persistent poor calls) and the effect this had on my daughter” (Parent 5). Parents also cited their own ‘child’s behavior’ (23.70%) and, to a lesser extent, their ‘child’s performances’ (14.81%) as prevalent stressors. The following quote from one mother illustrates how her daughter ‘not playing to full potential’ (13.33%) was a significant stressor:

My daughter is a technically very able player, quite athletic, and plays really well in her 1–1 lessons and squads. Then she gets onto court in a tournament, playing against someone who is clearly a casual, 2 h-a-week player (not 10 h like my daughter!), and my daughter plays “down,” doesn’t use her technique, and loses. I wonder if it is even worth her putting all the hours training, if that is what happens in tournaments! (Parent 112).

The presence of ‘other parents’ (15.56%) during competitions was another stressor and included lower order themes such as ‘bad behavior and attitude’ (6.67%), ‘interfering with play’ (5.19%), and ‘intimidating and aggressive behavior’ (3.70%). Parents reported specific examples such as: “Parents of other players providing guidance beyond acceptable encouragement” (Parent 111) and “abusive parents who shout at your child during the match” (Parent 65) as pertinent stressors associated with other parents. In addition, parents self-reported stressors that related to ‘watching a match’ (14.81%), particularly if they thought their child should win or would lose badly: “I find watching my son play a stressful experience, especially if I know the score…” (Parent 65). Parents also reported the ‘outcome of matches’ (11.85%) as a stressor, especially their ‘child losing a match’ (2.22%), ‘child’s reaction to match outcome’ (2.96%), and ‘consoling child/helping them to cope’ (5.19%). For instance, one parent stated: “If my child doesn’t win/play well he gets very upset and is sometimes physically sick. He thinks he’s letting everyone down… himself/me/coach” (Parent 53). Less frequently cited competition stressors included their ‘children’s psychological readiness to perform’ (6.67%) and ‘poor refereeing’ (2.96%).

#### Organizational Stressors

Six higher order themes, 25 low order themes (see Table 1), and 135 raw data themes referred to organizational stressors associated with children’s tennis involvement. A substantial number of participants mentioned ‘finances’ (25.19%) and particularly the ‘cost of coaching, tournaments, and travel’ (21.48%) as a source of stress. As one parent stated: “Finance. The cost of lessons, squads, competitions, and traveling to competitions” (Parent 11). Similarly, another parent added: “Financial – we have not had a family holiday for 2 years since my daughter started competing at a higher level!” (Parent 122). ‘Time’ (22.22%) was also a stressor for a large number of parents and, in particular, ‘limited family and partner time’ (9.63%) as demonstrated in the following quotes: “Lack of time. Tennis competitions (event time, traveling, and recently finding them) taking up too much family time” (Parent 11) and “competitions that are over two or more days present a constant problem for a family with more than one child, particularly where the other child does not play competitive tennis – it splits the family and creates rifts” (Parent 84). In terms of time related stressors, a small number of parents also appeared to be concerned about ‘work/tennis role conflict’ (2.96%), the ‘effect of unequal time spent on siblings’ (2.96%), and resented the time spent on tennis due to its negative ‘impact on social life/personal time’ (2.22%). One parent admitted: “I resent the way that the time (and money) involved in traveling to tennis squads and tournaments means my son is also put first leaving little time for anything I would like to be doing, and little money for anything else” (Parent 36).

Beyond the financial and time commitments of junior tennis participation, parents identified ‘coaching and training’ (16.30%) and ‘organizing bodies’ (14.81%) as stressors. Lower order themes included the ‘lack of recognition and support’ (5.93%), ‘pressure of the rating system’ (4.44%), ‘problems with talent identification system’ (2.22%), and general ‘disorganization and management issues’ (2.22%) as organizational stressors. The following quote captures parents’ frustration with the current rating system:

Too much emphasis for years on ratings, often non-reflective of ability, and until recently LTA refusal to acknowledge this. The LTA can’t see how the system is ‘abused’ by some players, and when you get your rating behind (e.g., through injury and player withdrawals) virtually no opportunity to catch up (Parent 14).

Other organizational stressors related to ‘tournaments’ (12.59%), and specifically ‘issues with entry, draws, and seedings’ (4.44%), ‘traveling to tournaments’ (3.70%), ‘poor organization/communication at tournaments’ (2.22%), ‘lack of umpire present’ (1.48%), and ‘tournament schedules’ (1.48%). As one parent wrote: “Referees being poorly organized, not getting players on, leaving long times in between matches, making mistakes in informing both players about starting times, not being friendly and helpful” (Parent 68). A small number of parents made reference to ‘injury’ related stressors (6.67%) and, in particular, ‘overuse injuries’ (3.70%). As one parent wrote: “My child has been constantly injured as a result of the amount of tennis training he has being taking part in” (Parent 41). Other parents cited ‘fear of injury’ (1.48%) and their ‘limited knowledge regarding injuries’ as stressors (1.48%).

#### Developmental Stressors

Six higher order themes, seven lower order themes (see Table 1), and 44 raw data themes referred to stressors associated with children’s development both within and outside of tennis. The most frequently cited developmental stressor within this dimension was their ‘child’s progress in tennis’ (16.30%). Specifically, parents referenced ‘selection pressures’ (6.67%), ‘progression relative to peers’ (4.44%), ‘tennis rating’ (2.96%), and ‘limited effort in training’ (2.22%) as lower order stressors. The following quote captures the concerns of parents in relation to these developmental factors: “I feel stressed about trying to keep my son’s ratings/rankings up with his peers/coaches’ expectations” (Parent 115). Furthermore, parents felt that ‘tennis decisions’ (7.41%) in relation to ‘coaching decisions’ (2.96%), ‘tournament decisions’ (2.96%), and ‘training decisions’ (1.48%) were key stressors. As one parent wrote:

I find it difficult to have to make choices and decisions about which tournaments my son should play and balancing out costs and aims from competition (i.e., good tough matches vs. easy points, not risking ratings losses, keeping up with other players who can travel further or fit in more tournaments vs. working on own goals) (Parent 36).

A small number of parents were concerned about the impact that tennis has on their ‘child’s education and social development’ (3.70%). As one parent wrote: “[Child’s name] spends 14 h a week doing sport outside school (tennis and football). I worry that this has an impact on both his school and his social development” (Parent 88). Beyond children’s development, some parents also reported stressors regarding their ‘child’s future in tennis’ (2.22%), the ‘impact of tennis on other sports/hobbies’ (1.48%), and their ‘child’s wellbeing and happiness’ (1.48%).

### Primary Appraisals

Of the total 342 separate self-reported stressors, 115 (33.65%) were appraised as a harm/loss, 113 (33.04%) were appraised as a challenge, 105 (30.70%) were evaluated as a threat, and 9 (2.63%) were evaluated as a benefit. Table 2 illustrates differences in the way competition, developmental, and organizational stressors were appraised. The results suggest that organizational stressors were most commonly appraised as harmful (40.00%) whilst threat appraisals were most commonly made in response to both developmental (43.18%) and competition (35.55%) stressors. Challenge appraisals were the second most frequently used appraisal across all general stressor dimensions whilst benefit appraisals were rarely made irrespective of the nature of the stressor.

**TABLE 2:** The appraisals and emotional profile for all stressors (*n* = 342) and general stressor dimensions (*n* = 334).

**General stressor**													
**dimensions**	**Appraisals**	**Frequency**		**Anxiety**		**Dejection**		**Excitement**		**Anger**		**Happiness**	
		**N**	***%***	***M***	***SD***	***M***	***SD***	***M***	***SD***	***M***	***SD***	***M***	***SD***
All		342	100	2.81	1.03	1.80	1.37	0.66	1.03	2.06	1.41	0.52	0.94
	Harm/loss	115	33.63	2.94	1.01	2.17	1.40	0.32	0.79	2.49	1.42	0.27	0.64
	Challenge	113	33.04	2.72	1.04	1.61	1.28	0.82^c^	1.04	1.76	1.35	0.70	1.03
	Threat	105	30.70	2.87	0.94	1.71	1.36	0.67	0.95	2.06	1.34	0.39	0.74
	Benefit	9	2.63	1.78	1.48	0.67	1.12	2.89	1.45	0.33	0.50	2.89	1.45
Competition		155	100	2.88	1.07	1.79	1.41	0.56	0.92	2.39	1.43	0.40	0.85
	Threat	52	35.55	2.85	1.06	1.50	1.35	0.77	0.94	2.31	1.38	0.42	0.75
	Challenge	51	32.90	2.90	0.99	1.76	1.26	0.63	0.96	2.14	1.50	0.51	0.95
	Harm/loss	49	31.61	2.96	1.15	2.16	1.59	0.16	0.47	2.86	1.31	0.12	0.39
	Benefit	3	1.94	2.00	1.73	1.00	1.00	2.33	2.08	0.67	0.58	2.67	2.31
Organizational		135	100	2.79	0.97	1.86	1.31	0.64	1.03	1.93	1.32	0.54	0.90
	Harm/loss	54	40.00	2.94	0.92	2.06	1.23	0.50	1.004	2.22	1.46	0.43	0.79
	Challenge	45	33.33	2.53	1.10	1.58	1.36	0.80	1.06	1.58	1.12	0.76	1.11
	Threat	34	25.19	2.91	0.79	1.94	1.30	0.53	0.90	2.00	1.23	0.35	0.60
	Benefit	2	1.48	2.00	1.41	1.50	2.12	2.50	2.12	0.50	0.71	2.00	1.41
Developmental		44	100	2.70	0.95	1.80	1.37	0.98	1.21	1.27	1.23	0.75	1.16
	Threat	19	43.18	2.84	0.90	1.89	1.45	0.63	1.07	1.47	1.26	0.37	0.97
	Challenge	14	31.82	2.50	1.02	1.36	1.08	1.57	1.02	0.86	0.95	1.21	1.05
	Harm/loss	9	20.45	2.78	0.83	2.67	1.22	0.22	0.67	1.78	1.39	0.22	0.67
	Benefit	2	4.55	2.50	2.12	0	0	3.50	0.71	0	0	3.50	0.71

**M, mean score; SD, standard deviation*.*

### Emotions

Parents reported moderate levels of anxiety and anger, low levels of dejection, and very low levels of excitement and happiness in relation to the stressors recalled (see Table 2). Due to low numbers, stressors categorized as other (*n* = 8) and appraisals categorized as benefit (*n* = 9) were not included in analyses, resulting in a sample of 327 (two of the other stressors were appraised as benefit). Therefore, the means reported in text vary compared to Table 2, which includes the full stressor, appraisal, and emotion profile. A 3 (stressor) × 3 (appraisal) MANOVA indicated a significant main effect for stressor, Wilks’ Λ = 0.91, *F*(10,628) = 3.09, *p* = 0.001, $\eta_{p}^{2}$ = 0.05, and appraisal, Wilks’ Λ = 0.88, *F*(10,628) = 4.03, *p* < 0.001, $\eta_{p}^{2}$ = 0.06, on experienced emotion. There was a non-significant interaction between stressor and appraisal, Wilks’ Λ = 0.94, *F*(20,1042) = 1.02, *p* = 0.436, $\eta_{p}^{2}$ = 0.02. Regarding the stressor categories, follow up comparisons indicated greater anger in competition stressors (*M* = 2.43 ± 1.42) compared to both developmental (*M* = 1.33 ± 1.22, *p* < 0.001, CIs: 0.47, 1.65) and organizational stressors (*M* = 1.95 ± 1.32, *p* = 0.006, CIs: 0.12, 0.89). In addition, in the analyses with the extreme cases removed, greater anger was reported in organizational compared to developmental stressors (*p* = 0.037, CIs: 0.03, 1.22). Other comparisons were non-significant. Regarding the appraisal categories, follow up comparisons indicated greater dejection (*M* = 2.15 ± 1.40, *p* = 0.005, CIs: 0.18, 1.28) and anger (*M* = 2.46 ± 1.43, *p* = 0.003, CIs: 0.21, 1.31) in harm appraisal compared to challenge (*M*_dejection_ = 1.64 ± 1.28, *M*_anger_ = 1.75 ± 1.35) appraisal. In addition, in the analyses with the extreme cases removed, greater dejection was reported in harm appraisal compared to threat (*M* = 1.67 ± 1.33) appraisal (*p* = 0.033, CIs: 0.04, 1.12). There was also greater excitement (*M* = 0.82 ± 1.04, CIs: −1.08, −0.33) and happiness (*M* = 0.70 ± 1.05, CIs: −0.91, −0.23, both *p* < 0.001) in challenge appraisal compared to harm (*M*_excitement_ = 0.33 ± 0.80, *M*_happiness_ = 0.28 ± 0.65) and threat appraisal (*M*_excitement_ = 0.67 ± 0.95, *p* = 0.038, CIs: −0.70, −0.02; *M*_happiness_ = 0.39 ± 0.74, *p* = 0.002, CIs: −0.75, −0.14). Other comparisons were non-significant. In sum, competitive stressors elicited greater anger compared to organizational and developmental stressors. Further, stressors appraised as harm elicited greater negative emotions (i.e., dejection and anger, but not anxiety) compared to challenge appraisals, while stressors appraised as a challenge elicited greater positive emotions (i.e., excitement and happiness) compared to both harm and threat appraisals.

### Coping Strategies

A total of 653 individual coping strategies were reported by parents, which were categorized into 79 lower order themes, 20 higher order themes, and three general coping dimensions (see Table 3). Seven entries were not classified because no strategies were reported as being used. General coping dimensions included: (a) mastery coping; (b) internal regulation; and (c) goal withdrawal coping.

**TABLE 3:** The general dimensions, higher order themes, and lower order themes of coping strategies including the frequency of parents (*n =* 135) reporting each strategy and coping effectiveness.

**General**	****				**Coping**	
**dimension**	**Higher order theme**	**Lower order theme**	**Frequency**		**effectiveness**	
			***N***	***%***	***M***	***SD***
Mastery	Communicating with child		66	48.89	5.93	2.30
		Discussing the situation	25	18.52	6.29	1.83
		Providing comfort and reassurance	13	9.63	5.81	1.94
		Providing advice and guidance	13	9.63	5.20	3.00
		Providing positive feedback	9	6.67	7.30	1.25
		Providing encouragement	7	5.19	4.27	1.74
		Confronting and discussing behavior	7	5.19	3.50	2.83
		Involving child in decision making	6	4.44	6.57	1.90
		Emphasizing performance over outcomes	4	2.96	6.00	2.31
		Displaying positive body language	3	2.22	8.67	1.15
		Providing feedback at an appropriate time	3	2.22	7.75	2.63
		Setting process goals	3	2.22	6.67	1.53
		Emphasizing enjoyment	2	1.48	7.00	1.41
	Information seeking		36	26.67	6.44	2.18
		Seeking information from child’s coach	25	18.52	6.50	2.39
		Researching information	6	4.44	6.75	1.58
		Seeking information from organizer	5	3.70	4.80	2.49
		Seeking information from physiotherapist	3	2.22	6.67	1.53
		Seeking information from strength and conditioning coach	2	1.48	7.50	0.71
		Seeking information from other parents	2	1.48	6.67	1.53
	Time management		33	24.44	6.59	2.03
		Planning, logistics, and being organized	20	14.81	6.39	1.99
		Selective tournament entry	9	6.67	6.90	2.38
		Scheduling time with siblings	6	4.44	6.33	2.34
		Sharing commitment with partner	6	4.44	8.22	1.20
		Scheduling family time	5	3.70	6.40	1.14
		Incorporating family trips and tennis	3	2.22	4.67	1.53
		Incorporating personal activities and tennis	1	0.74	4.00	0.00
		Training locally	1	0.74	8.00	0.00
	Financial management		25	18.52	6.11	2.09
		Budgeting	18	13.33	6.26	2.00
		Selective/limited tournament entry	5	3.70	5.00	2.10
		Setting up additional income	3	2.22	4.33	1.15
		Applying for funding	2	1.48	6.00	1.41
		Working full time	1	0.74	9.00	0.00
	Managing child’s tennis progress and development		22	16.30	7.41	1.84
		Scheduling/enforcing break from tennis	9	6.67	7.00	2.26
		Changing coach/training center	6	4.44	7.83	1.60
		Employing a sport psychologist	5	3.70	6.83	1.17
		Ensuring child completes rehab exercises	2	1.48	7.00	2.83
		Moving child abroad	2	1.48	8.50	2.12
		Scheduling regular meetings with child’s coach	1	0.74	8.00	1.41
		Changing physiotherapist	1	0.74	10.00	0.00
	Changing parenting behavior		20	14.81	5.72	2.73
		Concealing emotions	8	5.93	5.63	2.72
		Punishing child’s behavior	5	3.70	4.25	2.66
		Allowing child to make own choices	4	2.96	7.80	1.92
		Giving child space to calm down	4	2.96	6.25	2.75
	Reducing negative impact of others		11	8.15	6.93	1.71
		Influencing opponent’s parents	5	3.70	7.33	1.21
		Reducing negative impact of partner	5	3.70	7.00	2.00
		Maintaining presence courtside	2	1.48	5.5	2.12
	Involving the referee		11	8.15	4.64	2.73
	Preparation		9	6.67	4.50	2.59
		Preparing child mentally and physically for competition	7	5.19	5.00	2.67
		Planning communication with child	2	1.48	2.50	0.71
	Problem solving		8	5.93	6.22	2.22
	Overseeing child’s overall development		6	4.44	6.75	2.25
		Ensuring balance with school and other hobbies	6	4.44	7.29	1.80
		Monitoring child’s academic progress	1	0.74	3.00	0.00
Internal regulation	Cognitive reappraisal		46	34.07	6.41	2.08
		Placing stressor in perspective	15	11.11	5.78	2.10
		Focusing on the positives	14	10.37	6.60	1.73
		Rationalizing the situation	14	10.37	6.73	2.78
		Focusing on long term development	13	9.63	5.93	1.98
		Focusing on benefits of tennis participation	7	5.19	7.25	1.16
		Focusing on processes not outcomes	4	2.96	7.33	1.21
		Managing own expectations	4	2.96	5.25	0.50
	Seeking emotional support		26	19.26	6.15	1.68
		Talking about situation with other parents	11	8.15	5.94	1.24
		Talking about situation with partner/friend	10	7.41	6.83	1.80
		Talking about situation with multiple people	9	6.67	5.73	2.00
	Behavioral avoidance		26	19.26	6.62	2.15
		Watching match with a limited view/further away	9	6.67	7.33	1.66
		Avoiding contact with other parents	8	5.93	7.58	1.68
		Temporarily walking away	4	2.96	6.00	2.16
		Avoiding the LTA’s system/tournaments	4	2.96	6.00	2.94
		Avoiding contact with child	2	1.48	4.00	0.00
		Avoiding watching the match closely	2	1.48	3.50	2.12
		Avoiding contact with coach	1	0.74	5	0.00
	Emotional regulation		19	14.07	6.08	1.85
		Trying to keep calm	13	9.63	6.33	1.91
		Deep breathing	7	5.19	5.30	1.16
		Smoking a cigarette	1	0.74	10.00	0.00
	Distraction		13	9.63	6.47	2.20
		Distraction with another task during a match	8	5.93	7.11	1.96
		Distraction by talking to other parents	3	2.22	5.75	2.50
		General distraction	2	1.48	5.00	2.83
	Cognitive avoidance		12	8.89	4.54	2.44
	Acceptance		9	6.67	6.12	2.74
Goal withdrawal	Behavioral disengagement		28	20.74	5.31	3.05
		Not watching the match/training	13	9.63	4.77	3.09
		Walking away from the match/training	12	8.89	6.15	3.18
		Stopped child playing tennis	2	1.48	5.00	0.00
		Stopped entering certain events	1	0.74	2.00	0.00
	Venting emotions		12	8.89	4.00	2.85
		Complaining	9	6.67	3.92	3.06
		Arguing	2	1.48	3.00	0.00
		Crying	1	0.74	7.00	0.00
No coping			6	4.44	2.86	3.48

**M, mean score; SD, standard deviation*.*

#### Mastery Coping

Eleven higher order themes, 49 lower order themes (see Table 3), and 374 coping strategies were categorized as mastery coping (i.e., parents attempting to take control of a stressful situation and eliminate the stressor; Nicholls et al., 2016). The most frequently cited mastery coping strategy by parents was ‘communicating with child’ (48.89%), which primarily included ‘discussing the situation’ (18.52%). As one parent explained: “Constant talking to him after matches to help deal with these emotions and try to make him see that if he carried on trying then matches can be turned round” (Parent 128). Other lower order themes included ‘providing advice and guidance’ (9.63%) before matches and ‘providing comfort and reassurance’ (9.63%) after defeats. For instance, in relation to the stressor of their child’s opponent making bad line calls and cheating, one parent explained:

Prior to the match I try and get him to realize that there will always be dodgy [line] calls that he needs to focus on his own game and realize that he is handing the game to the other child if he lets it get to him. He needs to challenge the calls and call the referee over if it is too bad (Parent 53).

In addition to communicating with their child, parents regularly used ‘time management’ (24.44%) as a higher order coping strategy that consisted of ‘planning, logistics, and being organized’ (14.81%), ‘selective tournament entry’ (6.67%), ‘scheduling time with siblings’ (4.44%), and ‘sharing commitments with partner’ (4.44%). Similarly, parents also used ‘financial management’ (18.52%) as a coping strategy and, particularly, ‘budgeting’ (13.33%) as one mother self-reported:

Looking to set up a small business alongside part time work so we have more money coming in and still be flexible for coaching and tournaments. But I will miss out on seeing my daughter play and the tournaments will be down to her dad (Parent 122).

‘Information seeking’ (26.67%) was another frequently cited coping strategy, with a number of parents ‘seeking information from child’s coach’ (18.52%) as well as other key stakeholders (i.e., organizer, physiotherapist, strength and conditioning coach, and other parents). Some parents also used coping strategies such as ‘managing child’s tennis progress and development’ (16.30%), including ‘scheduling/enforcing a break from tennis’ (6.67%), ‘changing coach/training center’ (4.44%), and ‘employing a sport psychologist’ (3.70%) as explained in the following quote:

Engaged external professional help [sport psychologist] for our child and ourselves, utilize preparation routines and put our child at the center of the solution and recognize it is not a quick fix, give them the mechanisms and create an environment that will support them to help themselves supported by a team including ourselves as parents (Parent 110).

Less frequently cited higher order coping strategies included, ‘changing parenting behavior’ (14.81%), ‘reducing negative impact of others’ (8.15%), ‘involving the referee’ (8.15%), ‘preparation’ (6.67%), ‘problem solving’ (5.93%), and ‘overseeing child’s overall development’ (4.44%).

#### Internal Regulation

Two hundred and twenty-nine coping strategies, 23 lower order themes, and seven higher order categories (see Table 3) were characterized as referring to internal regulation (i.e., attempting to manage the internal responses to stress; Nicholls et al., 2016). Over a third of parents used ‘cognitive reappraisal’ strategies (34.07%), which referred to: ‘Placing stressor in perspective’ (11.11%), ‘focusing on the positives’ (10.37%), ‘rationalizing the situation’ (10.37%), ‘focusing on long-term development’ (9.63%), or ‘focusing on benefits of tennis participation’ (5.19%). The following quote illustrates this finding and how parents cope by focusing on the benefits of tennis participation:

I think about how much I love my children and how I want them to have as many different opportunities as possible. I think about the positive impact sport has had (and continues to have) on their self-esteem, confidence, motivation, determination, and discipline. They are aware of their bodies and how to stay healthy. I think about how sport has encouraged them to take risks, to try things, to learn how to cope with getting things wrong. They have benefitted from interacting with a wider circle of people through sport. I consider that the time I will get with them (of them wanting me around) is relatively short and I rationalize that I have plenty of time to treat myself later! Also, when I stop and add everything up and really think about it, I always come to the conclusion that even if I had more money I would spend it on them anyway! (Parent 11).

Parents also regulated their internal responses to stressors by ‘seeking emotional support’ (19.26%), which consisted mainly of ‘talking about situation with other parents’ (8.15%) or ‘talking about situation with partner/friend’ (7.41%). Other higher order themes included ‘behavioral avoidance’ strategies (19.26%), which included ‘watching match with a limited view/further away’ (6.67%), ‘avoiding contact with other parents’ (5.93%), and ‘temporarily walking away’ (2.96%). One parent explained why she watches with a limited view: “I sit so there is an obstruction in the way of the court to limit my view – again this stops me living every point” (Parent 50). Parents also used ‘emotional regulation’ (14.07%) coping strategies such as ‘trying to keep calm’ (9.63%) and ‘deep breathing’ (5.19%), as one parent disclosed: “If I go [to tournaments/matches] I try to take deep breaths and can’t wait for it to be over and go home” (Parent 87). Less frequently cited higher order coping strategies included ‘distraction’ (9.63%), ‘cognitive avoidance’ (8.89%), and ‘acceptance’ (6.67%). For instance, one parent explained how he charts matches (i.e., recording match statistics) as a distraction technique: “I have started charting matches for something to focus on and then share stats with him sometime after match as part of conversation about how he felt and what the stats say” (Parent 130).

#### Goal Withdrawal

Two higher order themes, seven lower order themes (see Table 3), and 43 individual coping strategies referred to goal withdrawal coping strategies (i.e., parents ceasing efforts to achieve a goal; Nicholls et al., 2016). Specifically, ‘behavioral disengagement’ (20.74%) consisted of lower order themes such as ‘not watching the match/training’ (9.63%). As one parent admitted: “I try to avoid taking him to matches and hope my husband will take my son. It actually makes me feel sick” (Parent 87). Other lower order coping strategies included ‘walking away from the match/training’ (8.89%) as one parent explained:

I removed myself from the match and walked away to get a coffee. [I was] angry and also feeling very concerned for my daughter who had done everything she is asked to when this happens but who was basically being bullied and cheated on court – disgraceful! Such inconsistency from official to official from tournament to tournament (Parent 3).

In a small number of cases, parents ‘stopped child playing tennis’ (1.48%). For instance, one parent wrote: “I supported my son but asked him to stop playing. The pressure was painful to watch and no one cares” (Parent 129). A small number of parents also attempted to cope by ‘venting emotions’ (8.89%). This higher order category was made up of lower order themes such as ‘complaining’ (6.67%), ‘arguing’ (1.48%), and ‘crying’ (0.74%).

### Coping Effectiveness

#### Mastery Coping Effectiveness

As seen in Table 3, within the mastery coping dimension (*n* = 374), the most effective higher order coping strategy was ‘managing child’s tennis development and progress’ (*M* = 7.41 ± 1.84), which included moderately effective lower order strategies such as ‘changing coach/training center’ (*M* = 7.83 ± 1.60), ‘scheduling/enforcing break from tennis’ (*M* = 7.00 ± 2.26), and ‘employing a sport psychologist’ (*M* = 6.83 ± 1.17). In addition, ‘reducing negative impact of others’ (*M* = 6.93 ± 1.71), ‘time management’ (*M* = 6.59 ± 2.03), and ‘overseeing child’s overall development’ (*M* = 6.75 ± 2.25) were perceived as moderately effective higher order strategies. Other higher order strategies such as ‘communicating with child’ (*M* = 5.93 ± 2.30) included a combination of moderately effective [e.g., ‘providing positive feedback’ (*M* = 7.30 ± 1.25)] and ineffective lower order strategies [e.g., ‘confronting and discussing behavior’ (*M* = 3.50 ± 2.83)]. Similarly, within the ‘changing parenting behavior’ (*M* = 5.72 ± 2.73) higher order theme, ‘allowing child to make own choices’ (*M* = 7.80 ± 1.92) was moderately effective, whilst ‘punishing child’s behavior’ (*M* = 4.25 ± 2.66) was considered to be a moderately ineffective strategy. ‘Involving the referee’ (*M* = 4.64 ± 2.73) and ‘preparation’ (*M* = 4.50 ± 2.59) were considered to be the most ineffective mastery higher order coping strategies.

#### Internal Regulation Coping Effectiveness

Turning attention toward internal regulation coping strategies (*n* = 229), ‘behavioral avoidance’ (*M* = 6.62 ± 2.15) was considered to be the most effective higher order strategy, with lower order strategies such as ‘avoiding contact with other parents’ (*M* = 7.58 ± 1.68) and ‘watching match with a limited view/further away’ (*M* = 7.33 ± 1.66) considered as moderately effective. Another higher order theme ‘cognitive reappraisal’ (*M* = 6.41 ± 2.08) was perceived as moderately effective, and included particularly effective lower order themes such as ‘focusing on benefits of tennis participation’ (*M* = 7.25 ± 1.16) and ‘focusing on processes not outcomes’ (*M* = 7.33 ± 1.21). Similarly, ‘seeking emotional support’ (*M* = 6.15 ± 1.68), ‘emotional regulation’ (*M* = 6.08 ± 1.85), ‘acceptance’ (*M* = 6.12 ± 2.74) and ‘distraction’ (*M* = 6.47 ± 2.20) were moderately effective higher order strategies and ‘distraction with another task during a match’ (i.e., charting, reading, and answering emails) was considered as a particularly effective lower order theme (*M* = 7.11 ± 1.96). In contrast, cognitive avoidance (*M* = 4.54 ± 2.44) was perceived to be moderately ineffective.

#### Goal Withdrawal Coping Effectiveness

Higher order goal withdrawal strategies (*n* = 43) such as ‘behavioral disengagement’ (*M* = 5.31 ± 3.05) was considered moderately effective. In contrast, ‘venting emotions’ (*M* = 4.00 ± 2.85) was perceived as a relatively ineffective coping strategy. Parents also considered ‘not watching the match/training’ (*M* = 4.77 ± 3.09) to be a moderately ineffective strategy and ‘complaining’ (*M* = 3.92 ± 3.06) was perceived as the least effective coping strategy within this coping dimension.

#### Overall Coping Effectiveness

Overall, the 646 coping strategies were considered by parents to be moderately effective (*M* = 6.10 ± 2.32). Due to low numbers, benefit appraisals (*n* = 15) were not included in statistical analyses, resulting in a sample of 631 strategies. Therefore, the means reported in text vary compared to Tables 3, 4. A 3 (stressor) × 3 (strategy) × 3 (appraisal) ANOVA indicated a non-significant main effect for stressor, *F*(2,606) = 0.85, *p* = 0.430, $\eta_{p}^{2}$ = 0.003 and appraisal, *F*(2,606) = 1.69, *p* = 0.186, $\eta_{p}^{2}$ = 0.01, on effectiveness. A significant main effect for strategy, *F*(2,606) = 6.01, *p* = 0.003, $\eta_{p}^{2}$ = 0.02, indicated greater effectiveness for mastery (*M* = 6.20 ± 2.27) and internal regulation (*M* = 6.15 ± 2.03) strategies compared to goal withdrawal (*M* = 4.77 ± 2.98). In terms of the two-way interactions, there was: (a) a significant interaction between stressor category and appraisal, *F*(4,606) = 3.69, *p* = 0.006, $\eta_{p}^{2}$ = 0.02; (b) stressor category and coping strategy, *F*(4,606) = 3.69, *p* = 0.006, $\eta_{p}^{2}$ = 0.02; and (c) a non-significant interaction between appraisal and coping strategy, *F*(4,606) = 0.27, *p* = 0.898, $\eta_{p}^{2}$ = 0.002. The three-way interaction between stressor category, appraisal, and coping strategy was non-significant, *F*(6,606) = 1.57, *p* = 0.154, $\eta_{p}^{2}$ = 0.02.

**TABLE 4:** The relationships between stressors, appraisals, coping strategies, and coping effectiveness (*n =* 646).

						**Internal**		**Internal**		**Goal**		**Goal**	
		**Mastery**		**Mastery**		**regulation**		**regulation**		**withdrawal**		**withdrawal**	
		**coping**		**coping**		**coping**		**coping**		**coping**		**coping**	
**Stressor**	**Appraisal**	**strategy**		**effectiveness**		**strategy**		**effectiveness**		**strategy**		**effectiveness**	
		***F***	***%***	***M***	***SD***	***F***	***%***	***M***	***SD***	***F***	***%***	***M***	***SD***
All	All	374	57.89	6.23	2.26	229	35.45	6.20	2.05	43	6.66	4.77	2.98
	Harm/loss	110	29.41	5.70	2.30	82	35.81	5.72	2.18	19	43.18	4.32	2.89
	Threat	125	33.42	5.90	2.32	71	31.00	6.01	1.83	17	39.53	4.65	3.08
	Challenge	132	35.29	6.89	2.03	68	29.69	6.82	1.89	7	16.28	6.29	2.93
	Benefit	7	1.87	7.86	1.07	8	3.49	7.38	2.33	0	n/a	0.00	0.00
Competition	All	164	43.85	5.81	2.42	113	49.34	6.27	2.07	28	65.12	4.96	3.01
	Harm/loss	45	27.44	5.11	2.43	41	36.28	5.73	2.24	10	35.71	3.60	2.63
	Threat	56	34.15	4.98	2.22	36	31.86	5.75	1.61	12	42.86	5.08	3.15
	Challenge	62	37.80	7.03	2.08	30	26.55	7.40	1.69	6	21.43	7.00	2.45
	Benefit	1	0.61	8.00	0.00	6	5.31	7.33	2.73	0	0.00	0.00	0.00
Developmental	All	52	13.90	6.31	2.14	33	14.41	5.88	1.65	2	4.65	4.50	4.95
	Harm/loss	9	17.31	5.44	1.74	5	15.15	4.80	2.28	0	0.00	0.00	0.00
	Threat	23	44.23	6.48	2.00	17	51.52	6.00	1.77	2	100	4.50	4.95
	Challenge	17	32.69	6.12	2.45	10	30.30	6.10	0.99	0	0.00	0.00	0.00
	Benefit	3	5.77	8.67	0.58	1	3.03	7.00	0.00	0	0.00	0.00	0.00
Organizational	All	158	42.24	6.63	2.05	83	36.24	6.23	2.18	13	30.23	4.38	2.90
	Harm/loss	56	35.44	6.21	2.17	36	43.37	5.83	2.14	9	69.23	5.11	3.10
	Threat	46	29.11	6.74	2.21	18	21.69	6.56	2.25	3	33.33	3.00	2.00
	Challenge	53	33.54	6.96	1.78	28	33.73	6.46	2.19	1	7.69	2.00	0.00
	Benefit	3	1.90	7.00	0.00	1	1.20	8.00	0.00	0	0.00	0.00	0.00

**F, frequency; M, mean score; SD, standard deviation*.*

The two interaction effects were followed up with adjusted pairwise comparisons. First, regarding stressor category and appraisal: (a) challenge, but not harm or threat, appraisals were managed more effectively for competition stressors (*M* = 7.20 ± 1.97, *n* = 96) compared to organizational (*M* = 6.72 ± 2.02, *n* = 85), stressors only (*p* = 0.035, CIs: 0.11, 3.98); and (b) competition, but not organizational or developmental, stressors were managed more effectively following challenge appraisals (*M* = 7.20 ± 1.97, *n* = 96) compared to both harm (*M* = 5.24 ± 2.43, *p* = 0.001, CIs: 1.31, 3.38, *n* = 95) and threat (*M* = 5.26 ± 2.17, *p* < 0.001, CIs: 0.89, 2.91, *n* = 104) appraisals.

Second, regarding stressor category and coping strategy: (a) mastery, but not internal regulation or goal withdrawal, coping was more effective for organizational (*M* = 6.60 ± 2.09, *n* = 160) compared to competition (*M* = 5.83 ± 2.43, *n* = 160), stressors only (*p* = 0.001, CIs: 0.28, 1.44); and (b) organizational, but not competitive or developmental, stressors were managed more effectively by mastery (*M* = 6.60 ± 2.09, *p* = 0.001, CIs: 1.14, 5.34, *n* = 160) and internal regulation (*M* = 6.21 ± 2.18, *p* = 0.004, CIs: 0.77, 5.06, *n* = 82) strategies compared to goal withdrawal (*M* = 4.38 ± 2.90, *n* = 13).

To summarize, irrespective of stressor category or appraisal, mastery and internal regulation coping strategies were more effective than goal withdrawal. Furthermore, with challenge appraisal, competition stressors were managed more effectively than organizational but not developmental stressors. Within competition, but not organizational or developmental stressors, challenge appraisal was linked to more effective stressor management than both harm and threat appraisal. Mastery coping, but not internal regulation or goal withdrawal, was more effective for organizational stressors compared to competition stressors, but not developmental stressors. Organizational, but not competition or developmental, stressors were managed more effectively through mastery and internal regulation strategies compared to goal withdrawal.

### Descriptive Relationships Between Stressors, Emotions, Coping, and Coping Effectiveness

The current study also provides initial insights into the descriptive relationship between parents’ stressors, emotions, coping strategies, and coping effectiveness (*n* = 646). Focusing first on the relationship between emotions, coping, and coping effectiveness, mastery coping strategies (*n* = 374) were considered to be the most effective coping strategy and were used when parents reported moderate levels of anxiety, low levels of dejection and anger, and very low levels of excitement and happiness (see Table 5). Parents who used internal regulation strategies (*n* = 229) also experienced moderate levels of anxiety, low levels of anger and dejection, and very low levels of excitement and happiness. In contrast, parents who used goal withdrawal strategies (*n* = 43) reported high levels of anxiety, moderate levels of anger and dejection, and very low levels of excitement and happiness (see Table 5).

**TABLE 5:** The relationship between competition, developmental, and organizational stressors, emotions, coping strategies, and coping effectiveness (*n =* 646).

**Stressor**	**Emotion**	**Mastery**					**Internal regulation**					**Goal withdrawal**				
			**Coping**		**Coping**			**Coping**		**Coping**			**Coping**		**Coping**	
			**strategy**		**effectiveness**			**strategy**		**effectiveness**			**strategy**		**effectiveness**	
		***F***	***M***	***SD***	***M***	***SD***	***F***	***M***	***SD***	***M***	***SD***	***F***	***M***	***SD***	***M***	***SD***
All	Anxiety	374	2.84	1.04	6.23	2.26	229	2.78	1.00	6.20	2.05	43	3.05	0.99	4.77	2.98
	Dejection	374	1.80	1.39	6.23	2.26	229	1.88	1.32	6.20	2.05	43	2.25	1.40	4.77	2.98
	Excitement	374	0.61	0.99	6.23	2.26	229	0.08	1.10	6.20	2.05	43	0.59	1.00	4.77	2.98
	Anger	374	1.99	1.39	6.23	2.26	229	1.98	1.40	6.20	2.05	43	2.75	1.48	4.77	2.98
	Happiness	374	0.44	0.87	6.23	2.26	229	0.65	1.03	6.20	2.05	43	0.43	0.93	4.77	2.98
Competition	Anxiety	164	2.90	1.10	5.81	2.42	113	2.95	1.01	6.27	2.07	28	2.97	1.15	4.96	3.01
	Dejection	164	1.91	1.46	5.81	2.42	113	1.87	1.38	6.27	2.07	28	2.00	1.46	4.96	3.01
	Excitement	164	0.46	0.79	5.81	2.42	113	0.71	1.06	6.27	2.07	28	0.79	1.15	4.96	3.01
	Anger	164	2.46	1.39	5.81	2.42	113	2.14	1.51	6.27	2.07	28	2.62	1.54	4.96	3.01
	Happiness	164	0.25	0.59	5.81	2.42	113	0.58	1.08	6.27	2.07	28	0.66	1.08	4.96	3.01
Developmental	Anxiety	52	2.94	0.92	6.31	2.14	33	2.24	0.79	5.88	1.65	2	3.00	0.00	4.50	4.95
	Dejection	52	1.60	1.42	6.31	2.14	33	1.79	1.24	5.88	1.65	2	3.00	1.41	4.50	4.95
	Excitement	52	1.15	1.24	6.31	2.14	33	0.79	1.05	5.88	1.65	2	0.00	0.00	4.50	4.95
	Anger	52	1.00	1.14	6.31	2.14	33	1.42	1.17	5.88	1.65	2	2.50	2.12	4.50	4.95
	Happiness	52	0.87	1.22	6.31	2.14	33	0.61	1.06	5.88	1.65	2	0.00	0.00	4.50	4.95
Organizational	Anxiety	158	2.74	1.01	6.63	2.05	83	2.76	1.01	6.23	2.18	13	3.23	0.60	4.38	2.90
	Dejection	158	1.76	1.31	6.63	2.05	83	1.95	1.29	6.23	2.18	13	2.69	1.18	4.38	2.90
	Excitement	158	0.59	1.04	6.63	2.05	83	0.93	1.18	6.23	2.18	13	0.23	0.44	4.38	2.90
	Anger	158	1.84	1.27	6.63	2.05	83	2.01	1.28	6.23	2.18	13	3.08	1.33	4.38	2.90
	Happiness	158	0.49	0.92	6.63	2.05	83	0.75	0.97	6.23	2.18	13	0.00	0.00	4.38	2.90

**F, frequency; M, mean score; SD, standard deviation.**

Table 5 also illustrates the descriptive relationships between competition, developmental, and organizational stressors, emotions, coping strategies, and coping effectiveness. For example, mastery (*n* = 164), internal regulation (*n* = 113) and goal withdrawal (*n* = 28) coping strategies were used when parents experienced moderate levels of anxiety and anger, moderate to low levels of dejection, and very low levels of excitement and happiness in response to competition stressors. Of these strategies, internal regulation was considered to be most effective. Mastery (*n* = 52) and internal regulation (*n* = 33) coping strategies were used when parents experienced moderate levels of anxiety; low levels of dejection and anger; low to very low levels of excitement, and very low levels of happiness when facing developmental stressors. Goal withdrawal strategies (*n* = 2) were used when parents experienced high level of anxiety and dejection and moderate levels of anger. Mastery coping strategies (*n* = 158) were used when parents reported moderate levels of anxiety, low levels of dejection and anger, and very low levels of excitement and happiness when facing organizational stressors. Internal regulation strategies (*n* = 83) were used when parents reported moderate levels of anxiety and anger, low levels of dejection, and very low levels of excitement and happiness. Finally, goal withdrawal strategies (*n* = 13) were used when parents experienced high anxiety and anger, moderate levels of dejection, and very low levels of excitement in relation to organizational stressors. Mastery coping strategies were the most effective way of coping with both organizational and developmental stressors.

## Discussion

The aim of this study was to build on the existing sport parent research through a thorough investigation of psychological stress among parents of competitive British tennis players. As such, the current study extends existing research on the stressors that parents experience (Harwood and Knight, 2009a, b; Burgess et al., 2016), the ways that stressors are appraised, the range of emotions experienced (Omli and LaVoi, 2012), and the coping strategies employed (Burgess et al., 2016). The discussion that follows outlines the contributions that the findings make to scientific understanding within each of these areas and integrates the qualitative and quantitative elements of this study to provide novel insights into psychological stress among sport parents. We also offer a number of recommendations for applied practitioners, coaches, and national governing bodies.

Focusing initially on the stressors that tennis parents experience, this study provides the largest investigation (*n* = 135) to date of the situations that British tennis parents appraise as taxing or exceeding their resources. In line with previous studies (i.e., Harwood and Knight, 2009a, b; Harwood et al., 2010; Burgess et al., 2016), parents in the current study reported experiencing a range of competition, organizational, and developmental stressors. The consistency of these findings across various youth sport contexts (i.e., tennis, gymnastics, and soccer) is concerning because research within developmental psychology has shown that parents who experience a greater numbers of stressors consistently display more negative parenting styles and behaviors (e.g., higher levels of disciplinary punishment and harsher interactions with their children; see Knight et al., 2009). These studies also suggest that limited progress has been made within the past decade to reduce the number of stressors that British tennis parents experience in youth sport (see Harwood and Knight, 2009a, b). Although recent headway has been made by the Lawn Tennis Association to address competition-related stressors (see LTA Tennis, 2017), there still remains a need for policy level changes to reduce some of the organizational and developmental stressors (i.e., finances, time, tournament structure, ratings system, and education-related concerns) that are difficult to address through educational approaches alone (Thrower et al., 2016).

Beyond identifying the stressors that parents experience in British tennis, the current study was the first to explore parents’ primary appraisals of self-disclosed stressors. The high frequency of negative appraisals (i.e., harm/loss or threat) in the current study is concerning given the implications that these types of appraisal have for parents’ experiences within youth sport contexts. Research with athletes has found positive associations between threat appraisals and mastery avoidance, performance approach, and performance avoidance goals as well as challenge appraisals and mastery goals (e.g., Adie et al., 2010; Nicholls et al., 2014). In addition, Ntoumanis et al. (2009) reported that individuals are more likely to appraise demands as a challenge as opposed to a threat or harm/loss when they feel autonomous and competent during a stressful encounter. Taking these points into consideration, it is possible that working with parents to alter their beliefs about what constitutes success in youth sport, develop more task oriented achievement goals for their child, and strengthen perceptions of parenting competence may increase the chance of parents making more adaptive primary appraisals (i.e., challenge or benefit; see Thrower et al., 2017). From a theoretical perspective, such approaches are likely to be particularly effective when combined with efforts to optimize parents’ secondary appraisals (Lazarus and Folkman, 1984; Lazarus, 1999) by, for example, helping parents to remain aware of the variety of coping options that they can use during stressful situations.

The present study was also the first to examine the range of emotions (i.e., pleasant and unpleasant) that arise during sport parents’ stress transactions. Findings add to existing sport parent research (i.e., Omli and LaVoi, 2012) by highlighting that parents experienced greater anger in relation to competition but not organizational or developmental stressors. Consistent with Lazarus (1999) suggestions, the findings presented here also illustrate that harm appraisal generated greater negative emotions (i.e., dejection and anger, but not anxiety) compared to challenge appraisal, whilst challenge appraisal generated greater positive emotions (i.e., excitement and happiness) compared to both harm and threat appraisal. These findings are consistent with the results of similar studies conducted with athletes (see Nicholls et al., 2011, 2012) and illustrate how primary appraisals play a crucial role in shaping the subsequent emotional responses and experiences of sport parents. Furthermore, developmental stressors (in comparison to organizational and competitive stressors) in the current study were most frequently appraised as a threat (i.e., future damage to goal commitment, values, or beliefs) and associated with the high levels of anxiety. Such findings suggest that the temporal nature of stressors (i.e., past vs. future) may influence not only the appraisal (i.e., damage already occurred vs. future damage) but also the specific type of emotion (e.g., anxiety, anger, dejection, excitement, and happiness) sport parents experience.

Building on the aforementioned points, our findings suggest that the emotions parents experience influence the coping strategies they select. For instance, parents in the current study tended to use internal regulation or mastery coping strategies when they experienced moderate to low levels of unpleasant emotions (i.e., anxiety, anger, and dejection) and goal withdrawal strategies when they experienced moderate to high levels of unpleasant emotions (i.e., anxiety, anger, and dejection). From a practical perspective, these findings suggest that reducing levels of unpleasant emotions (e.g., by encouraging gain, rather than loss, appraisals) may enable parents to select more adaptive coping strategies (i.e., mastery or internal regulation). Although the mechanisms influencing this proposed relationship are not fully understood, it may be that experiencing less unpleasant emotions (particularly anxiety) reduces cognitive interference and enables parents to select a more effective coping strategy (McCarthy et al., 2013).

Turning attention toward coping strategies, novel insights have been reached in this study regarding the most effective coping strategies parents used in response to competition, organizational, and developmental stressors. For example, our findings suggest that organizational stressors (but not competition or developmental stressors) were managed more effectively by mastery and internal regulation strategies when compared to goal withdrawal strategies. Further, when appraised as a challenge, competition stressors were managed more effectively compared to organizational, but not developmental, stressors. These findings are consistent with Lazarus and Folkman’s (1984) goodness-of-fit hypothesis of coping effectiveness, which proposes that coping strategies are most effective when matched to the level of controllability in any given situation. In line with transactional conceptualizations of stress (Lazarus and Folkman, 1984; Lazarus, 1999), coping strategies are not likely to be inherently effective or ineffective. Instead, it seems that coping effectiveness depends on the deployment of the most appropriate strategies at the right time (Knight and Holt, 2014). Some coping strategies (e.g., avoiding other parents) used by parents in the current study may, however, be viewed as maladaptive within the culture of British junior tennis, or may result in behaviors that are considered undesirable by young athletes (e.g., temporarily walking away; Knight et al., 2010). These suggestions are important for practitioners and should be taken into account when designing coping interventions for sport parents.

The current study and its applied implications should be considered in light of several limitations and insights that may enrich further research. First, only those stressors that were pertinent for parents at the time of data collection were explored. While this was a methodologically reasonable decision given the scope of this study, it does overlook the dynamic and recursive nature of stressors and stress more broadly. Future longitudinal research is needed to monitor stressors over time and build a more accurate and detailed picture of parents’ experiences. Use of diaries, think aloud protocols, or video assisted interviewing may assist in this respect. It would also be interesting to consider how parents’ level of experience or previous coping attempts influence their appraisals, emotions, and coping strategies. Second, the current study focused on primary appraising but secondary appraising is also an important part of stress transactions and one that can influence emotions and other outcomes (e.g., well-being, Lazarus, 1999, 2000). It was the complexity of analyses that were required to examine the components of stress transactions that meant secondary appraising was not investigated in the current work. Researchers are urged to consider methodologies that make possible more comprehensive explorations of appraising in sport. Third, although the parents in the current study identified multiple coping strategies, we did not explore the effectiveness of different combinations of coping strategies but, instead, focused on the effectiveness of each individual strategy. Future studies that examine the way in which multiple strategies are used together would progress this body of research and enable researchers to develop more effective coping interventions. Fourth, whilst sufficiently powered statistically, the investigation achieved a 9% response rate from the targeted parent population (*n* = 135). Challenges to engage parents with busy lifestyles were expected but researchers should carefully consider how to attract even more representative samples of a sport’s parent community in future work. Finally, although nomothetic methods have afforded greater understanding of tennis parents’ stress as a collective, more specific and idiosyncratic insights remain constrained. For example, exploring stress transactions among parents at different ages/stages of their child’s sporting journey (see Harwood and Knight, 2009a) would build on the contribution of the current study.

To conclude, this study used a mixed method design to provide unique insights to various components of psychological stress among parents of British tennis players. Furthermore, exploring the relationships and interactions between each stage of stress transactions provided a number of novel insights into the most effective ways of mediating the relationships between appraisal and emotions and of managing the emotions arising from a stressor (Lazarus, 1999). Such insights not only add to the sport parent literature but also provide crucial recommendations for practitioners, coaches, and national governing bodies who work with sport parents.

## Data Availability

The datasets generated for this study are available on request to the corresponding author.

## Ethics Statement

This study was carried out in accordance with the recommendations of the Loughborough University ethics committee with informed consent from all subjects. All subjects gave informed consent in accordance with the Declaration of Helsinki. The protocol was approved by the Loughborough University Ethics committee.

## Author Contributions

CH was responsible for the conceptualization and management of research program. FD contributed to the study design and planning. LF was responsible for data collection. ST and MS analyzed the data. CH, ST, FD, and MS were responsible for manuscript preparation.

## Conflict of Interest Statement

The authors declare that the research was conducted in the absence of any commercial or financial relationships that could be construed as a potential conflict of interest.
